# Bottom-up Filling of Damascene Trenches with Gold in a Sulfite Electrolyte

**Published:** 2019

**Authors:** D. Josell, T. P. Moffat

**Affiliations:** Materials Science and Engineering Division, National Institute of Standards and Technology, Gaithersburg, Maryland 20899, USA

## Abstract

Superconformal Au deposition is demonstrated in a Na_3_Au(SO_3_)_2 +_ Na_2_SO_3_ electrolyte using Bi species to catalyze the reduction of Au(SO_3_)_2_^3−^. Micromolar additions of Bi^3+^ to the sulfite-based electrolyte accelerate the reduction of Au(SO_3_)_2_^3−^ as shown by hysteretic voltammetry and rising chronoamperometric transients. Superconformal feature filling is observed over a defined range of Bi^3+^ concentration, potential and hydrodynamics. Over a more limited parameter range, approximately −0.9 V to −0.95 V, void-free, bottom-up filling of Damascene trenches is achieved. Furthermore, in the presence of significant convection the bottom-up filling is accompanied by passivation of the free surface. Bottom-up feature filling is characterized by a counterintuitive dependence of the free surface reactivity on the available flux of the Bi^3+^ accelerator species suggestive of an unusual coupling between hydrodynamic transport, shear and interfacial chemistry.

Superconformal film growth through additive-based electrodeposition underlies the successful implementation of Cu in state-of-the-art multilevel interconnect metallization for the silicon based semiconductor industry.^1,2^ Gold metallization for compound semiconductors and related optoelectronics^3,4^ that enable dense 3-D interconnect networks of arbitrary design and complex architecture will require Au Damascene processes and electroplating chemistries like those that provide void-free filling of recessed surface features for fabrication of Cu interconnects.^1^ Several Au-p-block metal additive systems have previously been associated with production of smooth, specular or bright surfaces.^5^ More recently, void-free superfilling of Damascene features has been demonstrated using Pb as a surfactant catalyst for Au deposition from KAu(CN)_2_ electrolytes^6,7^ in accord with the Curvature Enhanced Accelerator Coverage (CEAC) mechanism originally developed to describe feature filling in Cu additive systems. However, both the toxicity and the aggressive nature of the alkaline electrolyte toward photoresist materials represent serious shortcomings of the cyanide-based systems.^3,4^ Micromolar concentrations of Pb added to Na_3_Au(SO_3_)_2_ + Na_2_SO_3_ electrolyte also yield Au superfill of Damascene features through the CEAC mechanism.^8^ However, the Restrictions On Hazardous Substances (ROHS) Directive precludes the addition of Pb at even these low concentrations. In another approach sodium mercaptopropane sulfonate (MPS) added to a commercial pH buffered Na_3_Au(SO_3_)_2_ + Na_2_SO_3_ electrolyte acts as a suppressor that in combination with a Tl^+^ “grain refiner” enables superconformal deposition.^9^ Feature filling is consistent with leveling through an incorporation-derived gradient of suppressor concentration as suggested by electroanalytical measurements. Additive incorporation in the Au deposit implicit in the mechanism is a concern with this approach, as is the use of Tl^+^, again due to ROHS. A different suppressor, polyethyleneimine, exhibiting sharply defined suppression breakdown, manifest as S-shaped negative differential resistance (S-NDR) during voltammetry, yields superconformal Au filling in a sulfite electrolyte that is entirely localized within recessed through-silicon-vias (TSV).^10^ However, the deposits contain nanoscale porosity, due in part to additive incorporation that is, again, integral to the filling mechanism.

The coinage metals, Au, Ag and Cu, can be deposited superconformally using electrolyte-additive systems containing accelerators that enable the CEAC mechanism of superfill. In these systems, decrease of surface area during growth on concave surfaces increases the coverage of adsorbed accelerator and thereby the local deposition rate while the opposite occurs on convex surface segments. The CEAC mechanism thus yields accelerated filling of recessed (i.e., concave) features through positive feedback. Examples include the alkaline cyanide and near-neutral sulfite Au electrolytes noted above,^6–8^ along with Cu sulfate electrolytes^2,11–16^ and Ag cyanide electrolytes.^17–22^ Feature filling models based on the CEAC mechanism accurately capture the observed superconformal feature filling and, more generally, surface smoothing and stabilization of surface planarity.^23,24^

Near-neutral sulfite electrolytes for Au electrodeposition are environmentally superior to cyanide, with research on them reportedly dating back to 1842.^25^ They do suffer from disproportionation, SO_2_ formation, and/or dithionnite-based decomposition in acidic media.^3,4,26–28^ However, their instability can be mediated using additives such as ethylenediammine and/or electrolyte modification, i.e., thiosulfate, enabling operation to pH values as low as 4.0.^25–29^ Sulfite plating baths, like cyanide, exhibit constrained metal deposition kinetics that are at least partly associated with the formation of inhibiting complexes and related species on the surface. Significantly for superconformal deposition, in cyanide electrolytes this native inhibition is lifted by adsorbed Pb, Tl, Bi and Hg, yielding hysteretic voltammetry and depolarization during chronopotentiometry, and accordingly, the additives are classified as accelerators of Au electrodeposition.^5^ When optimized, their addition permits fabrication of smooth, bright Au films from both cyanide and sulfite electrolytes that can be explained through the CEAC mechanism.^3–5,25–45^

This work is a continuation of the earlier studies of superconformal Au deposition using Pb additive^8^ and, more broadly, of accelerating additives for superconformal filling through the CEAC mechanism in general. The utility of Bi as a catalyst for superconformal Au deposition in sub-micrometer trenches is explored in the Na_3_Au(SO_3_)_2_+Na_2_SO_3_ electrolyte. The electrochemistry of the system was studied by voltammetry and chronoamperometry under defined mass transport conditions and compared to feature filling behavior under similar conditions.

## Experimental

The Au deposition detailed in this work used a sodium gold sulfite electrolyte (Na_3_Au(SO_3_)_2_, Technic 25^1^) containing 2 troy ounces of Au per liter, equivalent to 0.32 mol/L, that was diluted to one-fourth its original concentration using 18 MΩ cm water. Deposition studies were conducted in a cell containing 40 mL of 0.080 mol/L Na_3_Au(SO_3_)_2_ + 0.64 mol/L of Na_2_SO_3_, the latter as supporting electrolyte. The bismuth additive was introduced by anodic dissolution of 99.999% pure Bi. The stated concentrations are based on the dissolution charge with the assumption of 100% efficiency and 3 equivalents, i.e. Bi^3+^. Thus, the stated concentrations represent an upper bound estimate for monomeric species. Dosing was accomplished by cycling the potential from −0.58 V to −0.4 V typically at 2 mV/s while monitoring the total charge. Concentrations obtained through incremental dosing yield behavior consistent with that obtained using a single charge cycle. The electrolyte pH was approximately 9.5 as determined using a pH electrode calibrated to buffer solutions of pH 7.0 and pH 10.0. Cyclic voltammometry and chronoamperometry were performed using a Au rotating disk electrode (RDE) of 1.0 cm diameter that was electroplated on the end of a Ag rod embedded in epoxy. The Au RDE was polished to 1200 grit SiC paper before each experiment and current densities were evaluated based on the nominal (geometric projected) area. Feature filling was studied using 3 mm × 11 mm fragments of patterned wafers having a 0.2 μm thick Au seed on the free surface and a lesser amount on the side walls and bottoms of the patterned trenches and vias. The patterned substrates were rotated during deposition by attaching them to one end of a Pt spindle, like a helicopter blade (the patterned surface facing upwards) to give definition to the metal ion and additive transport. The spindle was masked by electroplater’s tape to reduce non-specimen currents and associated potential drop in the cell. Based on the ≈1 cm distance between the rotational axis and the imaged features, a 200π rad/min (100 rpm) rotation rate corresponds to an estimated 10 cm/s flow rate over the surface. The electrolyte was at room temperature (≈23°C) during deposition. A Hg/Hg_2_SO_4_/saturated K_2_SO_4_ reference electrode (SSE), connected to the working electrode compartment via a fritted bridge filled with saturated solution of potassium sulfate, was used for all experiments. The platinum counter electrode was held in a frit-separated cell immersed within the main cell.

## Electrochemical Measurements on Planar Substrates

**Figure 1.**
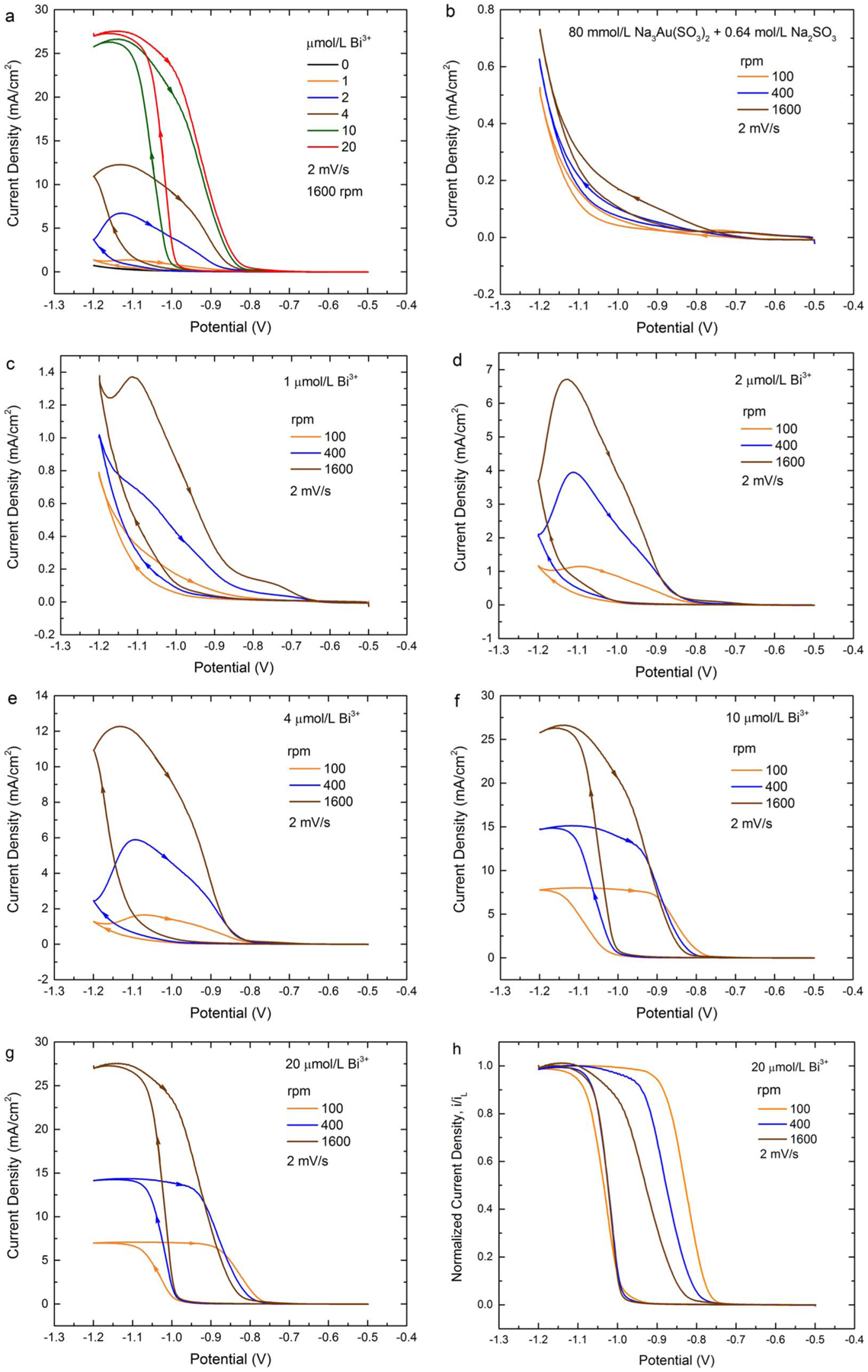
Cyclic voltammograms for 80 mmol/L Na_3_Au(SO_3_)_2_ + 0.64 mol/L Na_2_SO_3_ with the indicated Bi^3+^ concentrations and RDE rotation rates. a) Voltammograms showing the impact of Bi^3+^ concentration at rotation rate 1600 rpm. b-g) Voltammograms showing the impact of rotation rate at the indicated Bi^3+^. h) Voltammogram with current densities scaled by the plateau values at −1.1 V. For all, the applied potential was cycled from −0.5 V at 2 mV/s, deposition is at room temperature, and potentials are relative to SSE. Data was acquired using software compensation for 70% of the measured 7 Ω cell resistance (i.e., ≈2 Ω of uncompensated cell resistance). Cathodic currents are plotted positive.

Cyclic voltammetry on the Au RDE in 0.08 mol/L Na_3_Au(SO_3_)_2_ + 0.64 mol/L Na_2_SO_3_ electrolyte containing different Bi^3+^ concentrations is shown in Fig. 1. The dependence of the Au voltammetry on Bi^3+^ concentration is summarized in Fig. 1a with higher additive concentrations yielding increasing acceleration of the Au deposition rate at a given potential. Significant hysteresis on the return scan due to positive feedback between additive adsorption and the metal deposition is evident. Similar trends were evident as a function of hydrodynamics defined by the RDE rotation rate as shown in more detail in Figs. 1b–1g. In the absence of Bi^3+^ the RDE surface darkened slightly following the voltammetric “cycle (Fig. 1b); this roughening likely accounts for the slight hysteresis. Introduction of Bi^3+^ results in more significant hysteresis associated with its adsorption and acceleration of the metal deposition (Figs. 1c–1g). Substantial acceleration on the negative-going scan is evident at/and below −1.0 V. The acceleration is such that current continues to increase even on the positive-going, return scan from −1.2 V, to yield a negative differential resistance (NDR) regime indicative of positive feedback. This is especially clear for the electrolyte with 2 μmol/L Bi^3+^ and 4 μmol/L Bi^3+^. Visible smoothing of the RDE surface occurs during the voltammetric scan indicating that the large positive hysteresis is not due to an increase in surface area. An increase of rotation rate in the Bi-containing electrolytes substantially increases the current density at potentials negative of −1.0 V on the negative-going scan and up to −0.9 V on the positive-going scan. This sensitivity reflects the mixed control of the Au deposition reaction while the increase in current density with increasing Bi^3+^ concentration suggests a higher coverage of adsorbed Bi disrupts the native inhibition that otherwise constrains the reduction of Au(SO_3_)_2_^3−^. At higher Bi^3+^ concentration, (10 to 20) μmol/L, a further increase in Bi-catalyzed Au deposition is evident on the negative going scan. However, the deposition rate becomes diffusion limited on the return sweep as evident by the current plateaus in Figs. 1f and 1g. Levich-Koutecky analysis of the current density at −1.1 V for the 20 μmol/L Bi^3+^ solution gives a diffusion coefficient of 3.14 × 10^−6^ cm^2^/s for Au(SO_3_)_2_^3−^, assuming a kinematic viscosity of 0.01 cm^2^/s.^46^ Interestingly, as the potential approaches −0.9 V on the return scan an inversion occurs, with the current density at higher rotation rate decreasing more rapidly with potential. This is captured more clearly in Fig. 1h where the voltammetry has been normalized to the rotation rate-dependent limiting currents (from Fig. 1g). Under these conditions, the boost in the metal deposition kinetics is not strongly dependent on hydrodynamics. This may be because both Bi and Au are under similar mixed control or, alternatively, because the response is dominated by potential activation of the Bi accelerator whose coverage is fully established by the onset of the −1.0 V breakdown for 20 μmol/L electrolyte at all three rotation rates. In contrast, hydrodynamics exert a large effect on the return sweep with the reaction being more rapidly quenched at higher rotation rate. Increasing rotation rate is associated with a higher flux of the Bi^3+^ additive as well as a higher metal deposition rate. The increased rotation rate also corresponds to increased shear in the double layer adjacent to the interface. With regard to the last, previous work in cyanide electrolyte observed that variation in the hydrodynamics alters the chemical nature of the Bi overlayer.^5^ Likewise, recent work using vibrational spectroscopy challenges the widely assumed decoupling of mass transport and interface reaction kinetics, possibly relevant to the present system.^47^

**Figure 2.**
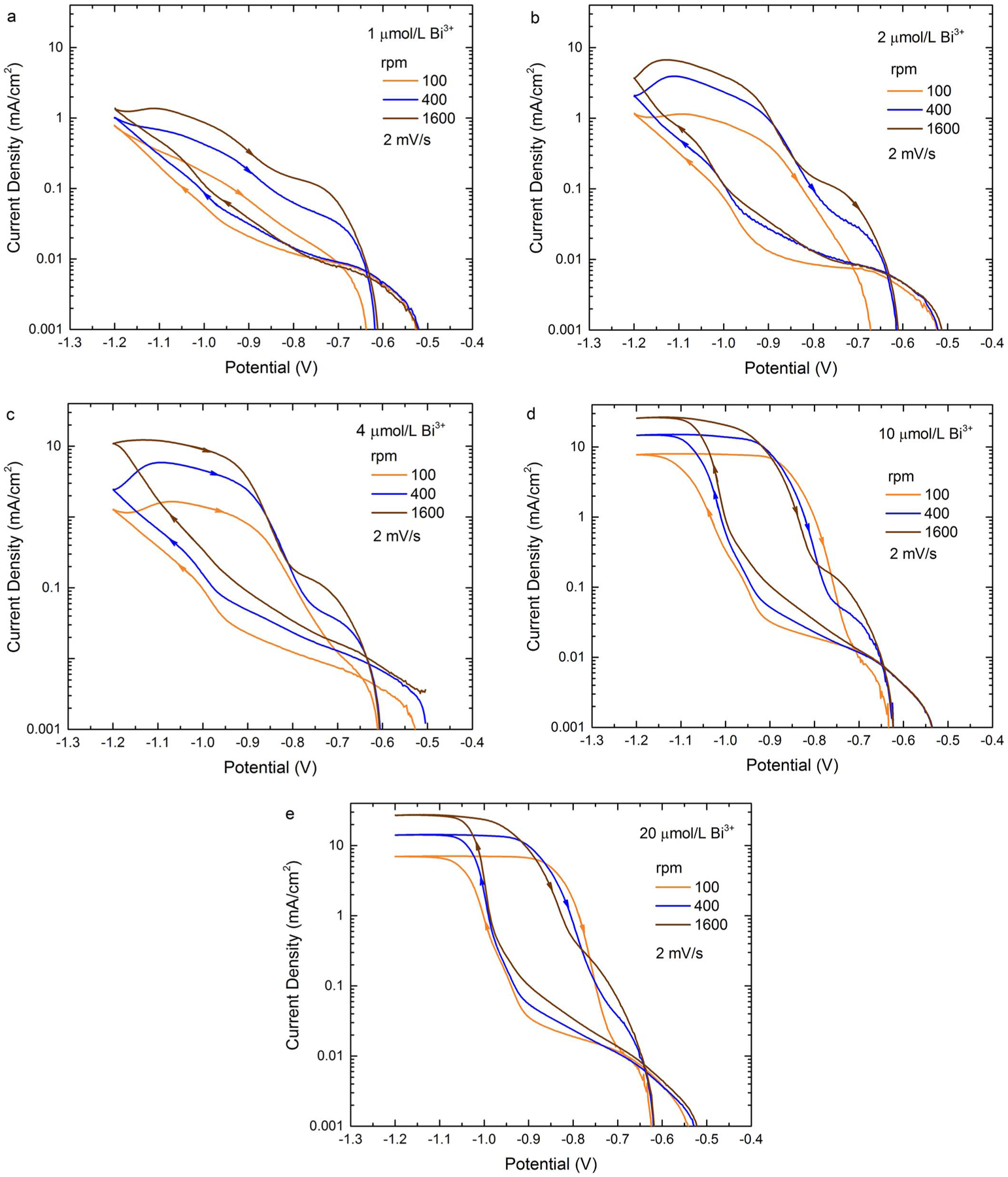
Voltammograms from Fig. 1 for Bi-containing electrolytes replotted to permit examination of lower current density. The applied potential was cycled from −0.5 V at 2 mV/s. Deposition is at room temperature, and potentials are relative to SSE. Data was acquired using software compensation for 70% of the measured 7 Ω cell resistance (i.e., ≈ 2 Ω of uncompensated cell resistance).

The voltammetric data for the Bi-containing electrolytes are replotted using a logarithmic scale in Fig. 2 to more clearly reveal the behavior in the low current region. The well-defined change in slope on the negative-going scans marks suppression breakdown. The re-assertion of suppression on the return scans positive of −0.9 V for 10 μmol/L Bi^3+^ and 20 μmol/L Bi^3+^ clearly occurs more rapidly at higher rotation rates, highlighting the behavior noted in the linear i-V plots. This is followed by a second inversion at more positive potential that is likely related to transport limited parasitic O_2_ reduction in the un-sparged electrolyte.

**Figure 3.**
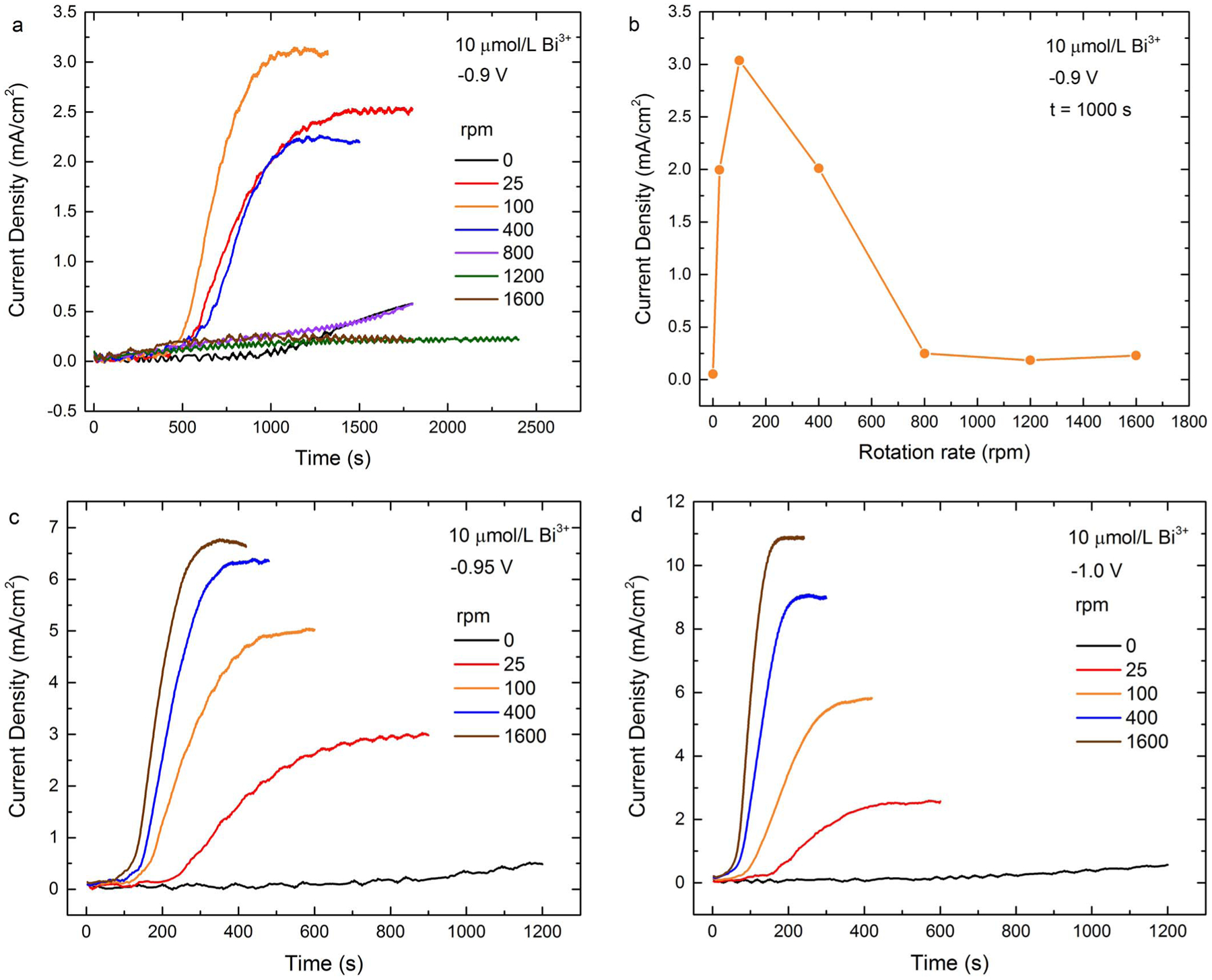
Chronoamperometric transients for 10 μmol/L Bi^3+^ at the stated potentials and RDE rotation rates. a) At −0.90 V the RDE remains passivated at the higher RDE rotation rates. b) The current density of the data in a) at 1000 s as a function of rotation rate. At c) −0.95 V and d) −1.00 V the timescale of the transients decreases and the magnitude increases with increasing rotation rate. Data was acquired without compensation for the 7 Ω measured cell resistance.

Chronoamperometry was used to more closely investigate the dynamics associated with the hysteretic voltammetric region for 10 μmol/L Bi^3+^, including the impact of potential and RDE rotation rate. The currents at −0.9 V exhibit an unusual non-monotonic dependence on hydrodynamics as shown in Fig. 3a. Following immersion and stepping of the potential from the open circuit condition to −0.9 V the current density remains below 0.2 mA/cm^2^ for several hundred seconds. This is consistent with the low values observed on the negative-going voltammetric sweep in Fig. 1f. Beyond 500 seconds the current begins to rise significantly, the rate and magnitude increasing with rotation rate up to 100 rpm. The rising current transients suggest a vertical trajectory across the hysteretic region of the voltammogram. However, increasing the rotation rate to 400 rpm does not increase the deposition reaction to the same extent, and for higher rotation rate of 800 rpm and above a complete inversion occurs and the metal deposition reaction fails to activate. The plot of current density at 1000 s as a function of rotation rate in Fig. 3b captures the non-monotonic dependence of the metal deposition on hydrodynamics. At more negative potentials, the unusual behavior is not observed, rather a monotonic trend of increasing deposition rate with hydrodynamics as seen in Figs. 3c and 3d. The current density increases to a plateau, the rise time decreasing and the steady-state current increasing with Bi^3+^ concentration. The decrease of rise time with rotation rate is congruent with acceleration due to transport limited Bi^3+^ accumulation while the steady state plateau value reflects limitation associated with transport of Au(SO_3_)_2_^3−^ to the electrode. In brief, at both −0.95 V and −1.0 V the transients reflect acceleration of the Au deposition reaction by Bi^3+^ accumulation, the acceleration increasing with coverage. For all specimen examined at nonzero rotation rate, the RDE surface was visibly smoother after deposition in the Bi-containing electrolyte, as with the voltammograms, verifying that the rising currents do not reflect increased surface area.

**Figure 4.**
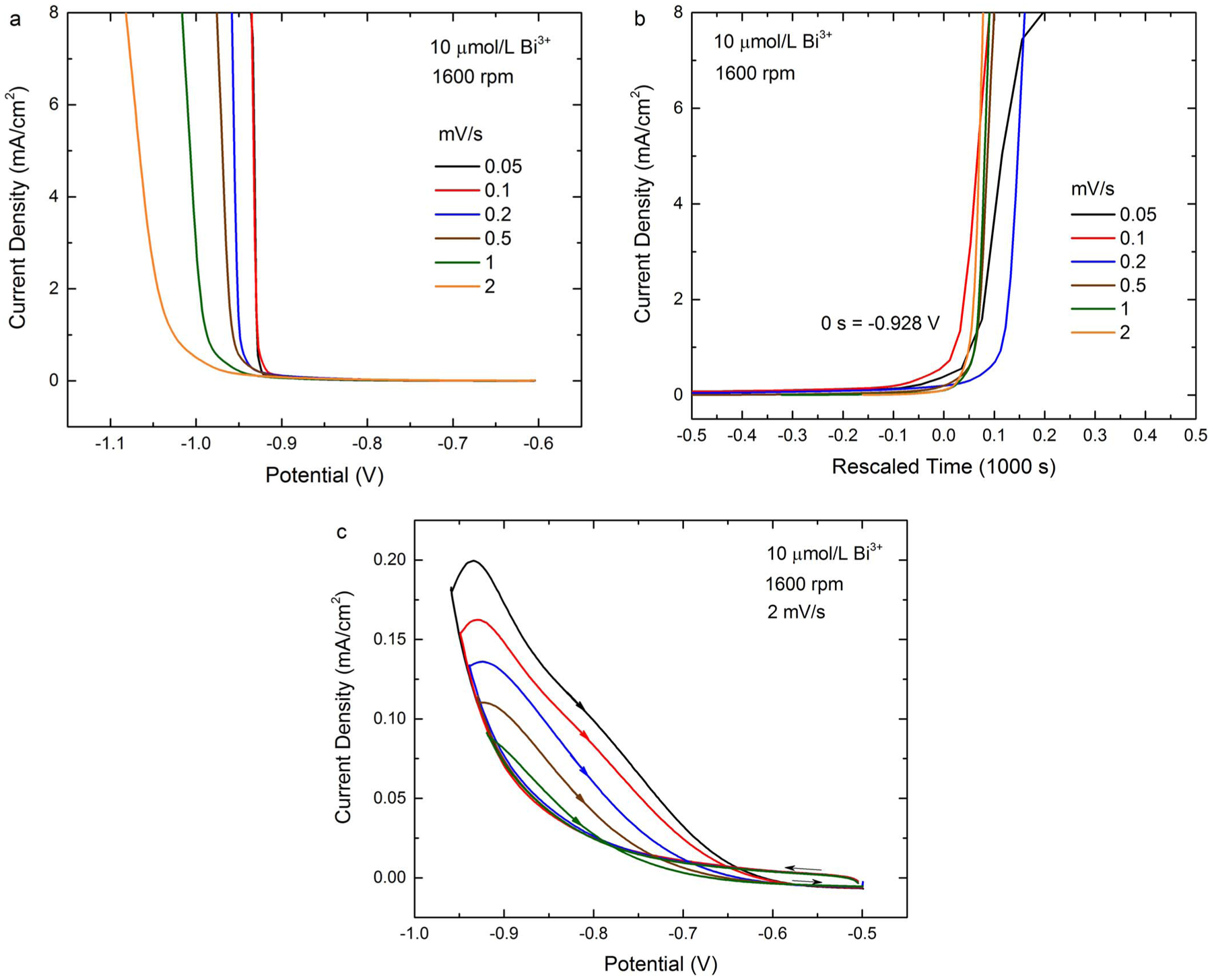
Voltammograms for 10 μmol/L Bi^3+^ and RDE rotation rate of 1600 rpm. a) Linear voltammograms with the applied potential scanned from −0.5 V at the indicated rates. b) Data from the same voltammograms plotted against the time after the potential reached −0.928 V, i.e., -(potential(V) + 0.928) divided by scan rate(V/s). c) Cyclic voltammograms with switching potential from −0.92 V to −0.96 V, all with scan rate 2 mV/s. Depositions are at room temperature, and potentials are relative to SSE. Data was acquired using software compensation for 70% of the measured 5.6 Ω cell resistance (i.e., leaving ≈1.7 Ω of uncompensated cell resistance).

The scan rate dependence of the voltammetry was also examined, and a sharpening of the suppression breakdown is evident at slower rates as shown in Fig. 4a. For the two slowest scan rates the breakdown thresholds, marked by a nearly vertical current rise, are almost indistinguishable, and the inverse-slope of ≈ 0.6 Ω represents a significant fraction of the 1.7 Ω uncorrected portion of the cell resistance (i.e., 30% of the measured 5.7 Ω impedance). Post experiment correction indicates the presence of a negative differential resistance, NDR, behavior that is obscured by the uncompensated cell impedance and associated with breakdown processes at potentials slightly negative of −0.9 V. Replotting the data in terms of the time elapsed before/after a threshold potential of ≈ −0.93 V (Fig. 4b), suggests that progression of suppression breakdown is primarily time-dependent. Variation of the voltammetric switching potential was used to further explore this matter. As shown in Fig. 4c, for switching potentials at or negative of −0.92 V the deposition rate on the return scan continues to increase as the scan rate goes positive (i.e., NDR). After a brief period, the current begins to decrease but the hysteresis persists to nearly −0.6 V, consistent with the broader ranging voltammograms shown in Fig. 1. The increase in positive feedback with more negative switching potential captured in Fig. 4c is consistent with the progressive activation of the surface associated with the longer lasting excursions negative of the critical potential.

While the data in Fig. 1 shows that Bi^3+^ adsorption is necessary for the lifting of suppression, Figs. 1, 2 and 4c show that once suppression has been at least partially lifted by scanning to or below −0.93 V the accumulating Bi^3+^ provides positive feedback, with the current density remaining elevated over a range of potentials extending to substantially more positive values.

**Figure 5.**
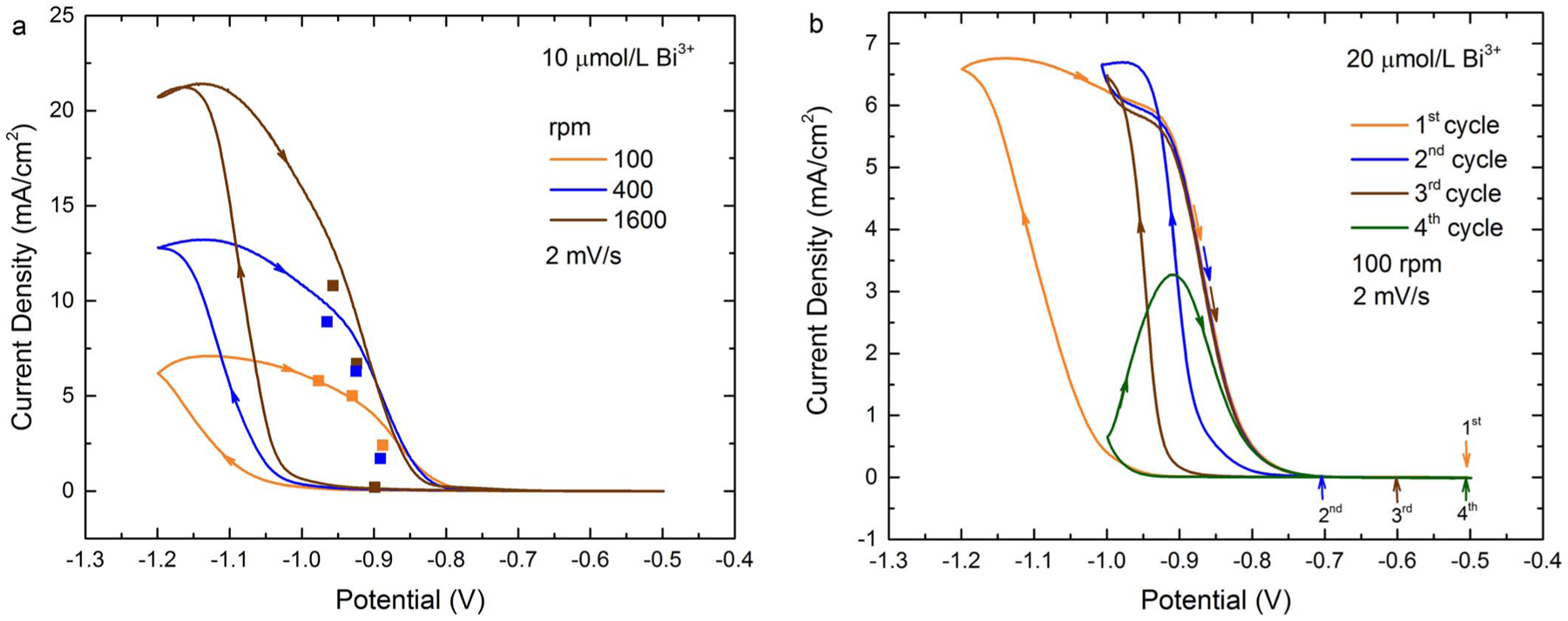
Cyclic voltammograms for the indicated Bi^3+^ concentrations and RDE rotation rates. The applied potential was cycled from −0.5 V at 2 mV/s. a) Cyclic voltammograms with average current density from the final 20 s of the plateaus in the chronoamperometry in Fig. 3 overlaid with the potentials iR compensated using a 5 Ω impedance to match that used to acquire the CVs (e.g., the 10.8 mA/cm^2^ plateau current density, I = 8.5 mA using RDE area, at −1.00 V applied potential and 1600 rpm is offset by i · R = 43 mV to −0.957 V). b) Multicycle voltammogram with the first to third cycles terminating successively at −0.7 V, −0.6 V and −0.5 V on the reverse (positive) scan and the second to fourth cycles beginning immediately thereafter at those potentials; starting points of cycles are indicated. The switching potential at the negative extreme is −1.2 V for the first cycle and −1.0 V for successive cycles. The RDE was rotating at 100 rpm. Depositions are at room temperature, and potentials are relative to SSE. Cathodic currents are plotted positive.

A comparison between voltammetry and chronoamperometry for 10 μmol/L Bi^3+^ is shown in Fig. 5a, where the average current densities for the final 20 s of the steady-state chronoamperometry (Fig. 3) are overlaid on the corresponding voltammograms. The chonoampero-metric transients were collected immediately following the voltammetry in the same electrolyte. The potentials associated with chronoamperometric data (uncompensated) are adjusted to reflect the use of partial compensation in the voltammetric experiments shown in Fig. 5a. The steady-state current densities at applied potentials of −1.00 V and −0.95 V are consistent with the values on the return scans of the voltammetry. For −0.90 V, the steady-state current densities for 100 and 400 rpm fall slightly below the return scans of the voltammetry while for 1600 rpm the surface is in the suppressed state, consistent with the negative-going voltammetric scan. Differences in the voltammograms in Fig. 5a from the nominally identical voltammograms in Fig. 1f suggest some unresolved variability in the charge-based additive dosing. The same net oxidative charge was passed through the bulk metallic Bi electrodes in each case although the area of the electrode used to charge the electrolyte in Fig. 5a was significantly smaller.

The potential-dependent deactivation of the Bi-activated electrode was examined by varying the upper potential limit during multicycle voltammetry. The four voltammograms are shown in Fig. 5b. The upper bounds for the first three cycles were −0.7 V, −0.6 V and −0.5 V, respectively. For the first cycle the negative switching potential was −1.2 V, and the voltammogram captures the full hysteretic region. After reaching −0.7 V the scan direction was reversed to begin the second cycle. After a small negative shift, the current increases, following more closely the active state captured in the return scan of the 1^st^ cycle. On the return sweep the system achieves the accelerated return of the first cycle. This result indicates that much of the Bi catalyzed character established on the 1^st^ cycle is maintained after cycling to −0.7 V. The upper limit after the second cycle was extended to −0.6 V. The higher starting potential for the 3^rd^ cycle leads to a larger negative shift in the onset of accelerated Au deposition, indicating more significant deactivation of the Bi catalyzed electrode occurred upon cycling to −0.6 V. Nevertheless, the return scan recovers the active state observed for the first two cycles. Once the third cycle reached −0.5 V the final cycle began. The trend of further delayed acceleration with more positive upper bound is maintained, acceleration being delayed almost to that associated with the negative scan in the original cycle. The sequence indicates progressively greater deactivation of the Bi accelerator occurs as the upper potential is shifted to more positive values, from −0.7 V to −0.6 V, with almost complete removal accomplished by cycling to −0.5 V.

## Feature Filling

**Figure 6.**
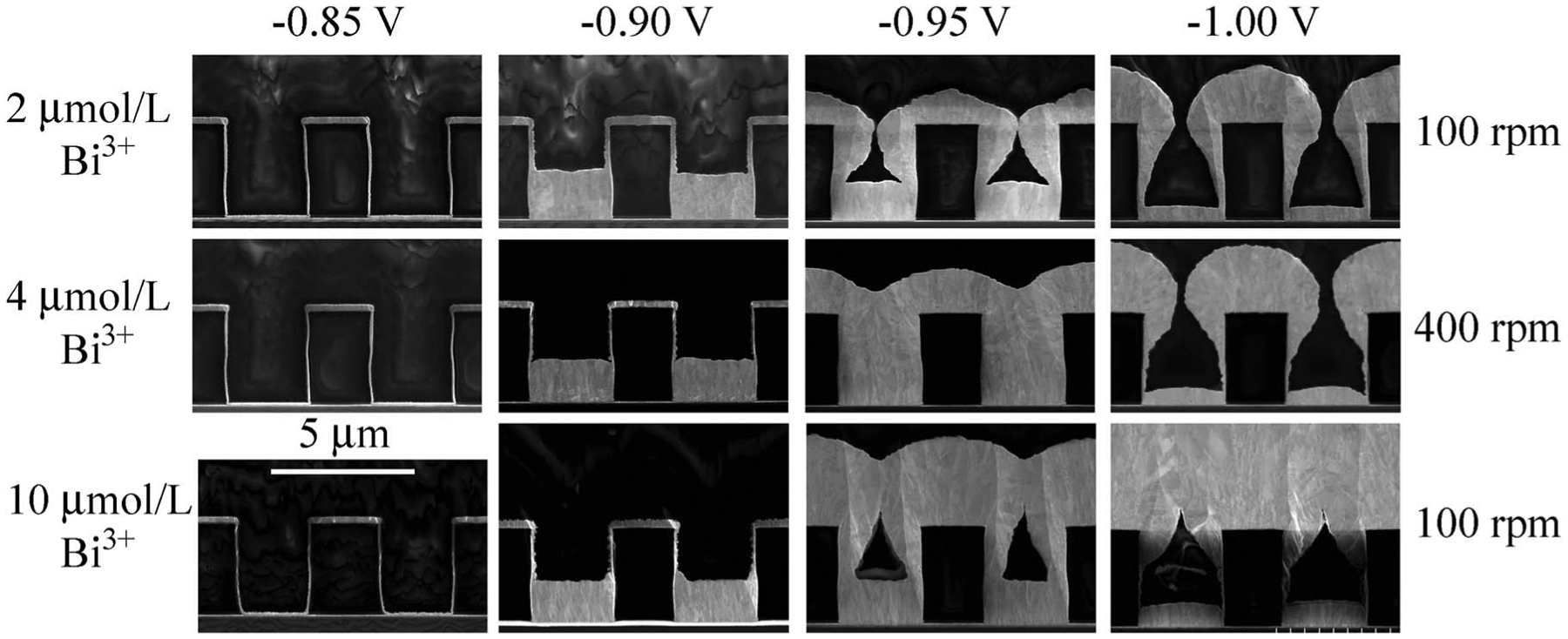
Gold deposition in trenches at potentials capturing the range of observed deposition behaviors, from full passivation to bottom-up deposition yielding void-free filling, and truncated bottom-up and almost entirely subconformal depositions yielding voided filling. Applied potentials, Bi^3+^ concentrations and rotation rates are as indicated. Deposition times were 20 min with 2 μmol/L Bi^3+^ and 10 min with both 4 μmol/L Bi^3+^ and 10 μmol/L Bi^3+^ aside from the (passive) depositions at −0.85 V, which were 30 min and 20 min, respectively.

Gold deposition on patterned trenches was examined as a function of Bi3+ concentration and applied potential, and a wide variety of behaviors were observed as shown in Fig. 6. Congruent with expectation derived from the electroanalytical experiments no metal deposition is evident at −0.85 V; the cross sections provide a useful reference for the Au seed layer metallization that covers all the surface segments that comprise the trench. In contrast, stepping to −0.9 V yields the onset of highly localized metal deposition with growth that is essentially restricted to the bottommost surfaces of the patterned array. For 2 μmol/L Bi^3+^ at 100 rpm bottom-up growth results in the trench being half filled in 20 min while similar filling with both 4 μmol/L at 400 rpm and 10 μmol/L Bi^3+^ at 100 rpm is obtained after 10 min. At the more negative potential of −0.95 V truncated bottom-up deposition with subconformal sidewall deposits gives rise to voided filling with 2 μmol/L Bi^3+^ and 10 μmol/L Bi^3+^ while at the intermediate 4 μmol/L Bi^3+^ concentration void-free, largely bottom-up filling of the trenches is evident. The differences in feature filling appear to correlate with the rotation rate, increased transport evidently enabling void-free filling. At −1.00 V, all three conditions yield subconformal deposition although, even here, there is a modest bottom-up filling component. The voids are the result of Au(SO_3_)_2_^3−^ depletion due to rapid deposition on the available surface.

**Figure 7.**
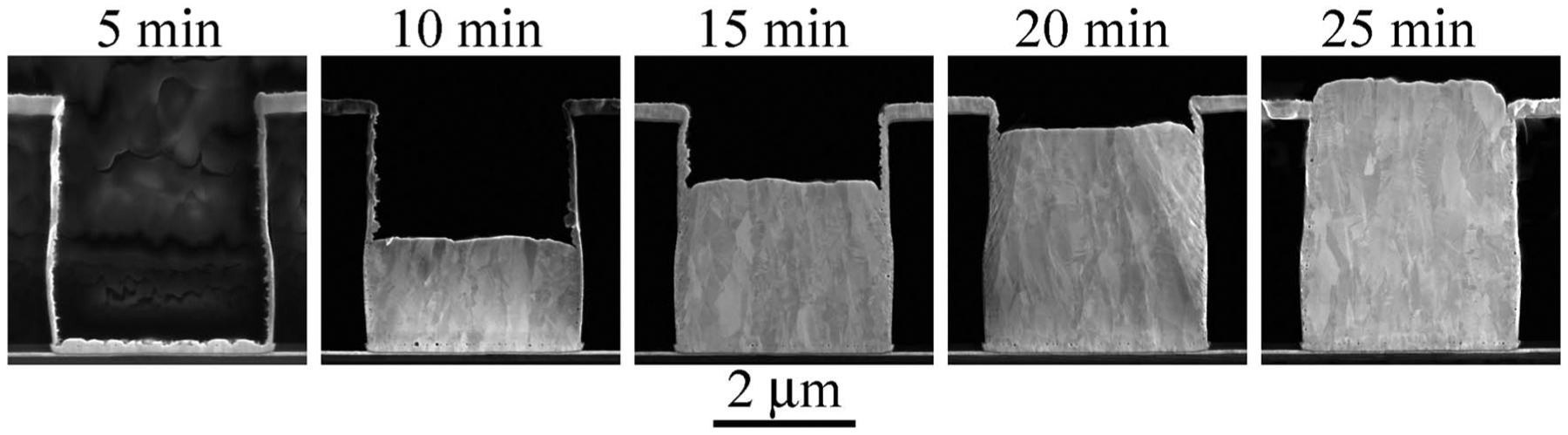
Au deposition in trenches at the indicated deposition times. The sequence captures the evolution of filling in electrolyte containing 4 μmol/L Bi^3+^ at applied potential of −0.90 V and substrate rotation rate of 400 rpm.

A closer look at the temporal evolution of deposition at −0.9 V is shown in Fig. 7. The sequence reveals complete bottom-up filling. An extended period of universal suppression precedes the bottom-up filling, with only the smallest hint of deposition being evident on the trench bottom at 5 min. Thereafter the bottom growth front advances at a steady rate of ≈ 2.5 nm/s that corresponds to a local current density of ≈ 2.4 mA/cm^2^. Close examination of the side walls reveals segments of irregular growth, but the sidewalls and free surface remain essentially inactive during feature filling.

**Figure 8.**
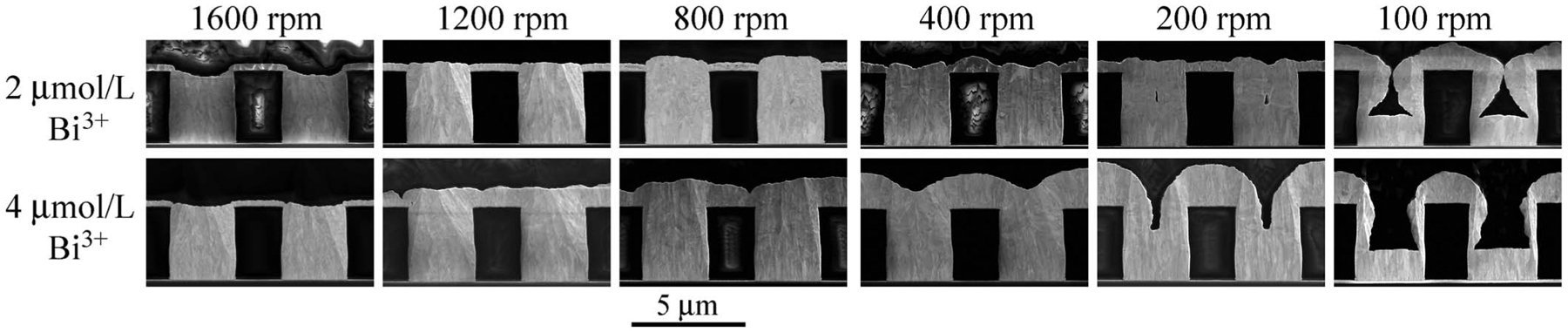
Gold deposition at −0.95 V for the indicated Bi^3+^ concentrations as a function of the substrate rotation rate. Deposition times are 20 min in the electrolyte containing 2 μmol/L Bi^3+^ and 10 min in the electrolyte containing 4 μmol/L Bi^3+^. Deposition on the free surface, fully passivated at the highest rotation rate, accelerates as the rotation rate decreases. Feature filling is impacted at the lowest rotation rates, apparently through associated metal ion depletion.

The impact of electrolyte hydrodynamics at −0.95 V implicit in Fig. 6 was examined explicitly for rotation rates between 100 rpm and 1600 rpm with 2 μmol/L Bi^3+^ and 4 μmol/L Bi^3+^. As shown in Fig. 8 bottom-up trench filling is obtained at 1600 rpm with deposition on the free surface entirely suppressed. Suppression of the free surface is lost as the rotation rate decreases, with deposition on the free surface reaching that inside the features at the lowest rotation rate. Enhanced deposit thickness on the bottom surface at 100 rpm suggests truncation of bottom-up growth through the subsequent onset of growth on the sidewalls and free surface. Keyhole-shaped voids indicate substantial Au depletion is associated with the deposition on the active free surface and sidewalls.

**Figure 9.**
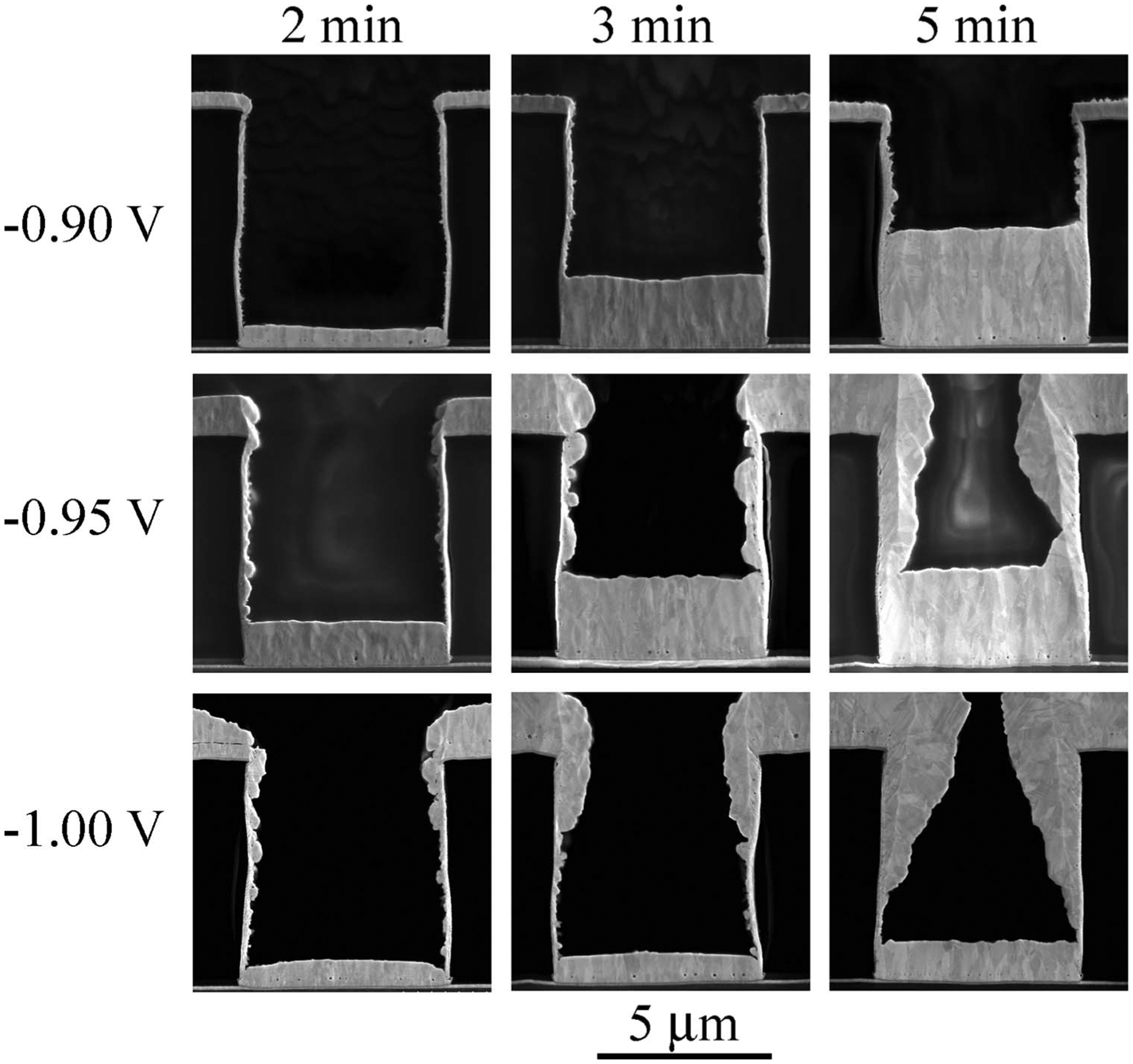
Gold deposition at the indicated potentials and deposition times. Filling with 20 μmol/L Bi^3+^ at substrate rotation rate 100 rpm.

To speed up the deposition process, trench filling was examined in 20 μmol/L Bi^3+^. The evolution of feature filling at −0.9 V, −0.95 V and −1.0 V and 100 rpm is shown in Fig. 9. As with the more dilute Bi^3+^ concentration in Fig. 7, bottom-up filling occurs at −0.90 V, although the growth rate of ≈8 nm/s substantially exceeds that obtained with only 4 μmol/L Bi^3+^ and 400 rpm. The local deposition rate of trench filling on the helicopter blade shaped specimen corresponds to ≈7.4 mA/cm^2^, which is close to the global diffusion limited value for Au deposition determined on the planar RDE (Fig. 1g) rotating at 100 rpm. The different geometries and flow fields preclude detailed analysis. However, they suggest a correspondence between deposition on the bottom surface of the trench and the return voltammetric scan and between the passive free surface and sidewalls and that of the negative going voltammetic scan. The limited and irregular growth visible at various points on the free surface and side walls does not interfere with the bottom-up growth dynamic. In contrast, 2 min at −0.95 V yields similarly accelerated deposition on both the bottom and free surfaces; only the side wall remains (relatively) suppressed. At this point the deposit on the bottom is approximately twice the thickness of that at −0.90 V. By 3 min the deposits on the bottom and free surfaces substantially thicken, but the sidewalls also become much more active. This sidewall expansion yields a sub-conformal, reentrant profile due to depletion of the Au(SO_3_)_2_^3−^ by 5 min, with growth at the bottom having effectively ceased. Deposition at −1.0 V, also exhibits accelerated deposition on the bottom and free surfaces at 2 min, with thicker deposits on the latter, while irregular growth on the side wall is already significant. Deposition on the upper sidewalls accelerates over the next minute while deposition on the bottom surface is minimal. A smooth but reentrant profile is established at 5 min due to depletion of the Au(SO_3_)_2_^3−^. The potential dependent transition from void-free, bottom-up growth to voided subconformal filling is accompanied by a corresponding transition from negligible deposition on the free surface between trenches at −0.90 V to deposition of approximately 1.5 μm at −0.95 V and 2 μm at −1.00 V at 5 min (not shown).

**Figure 10.**
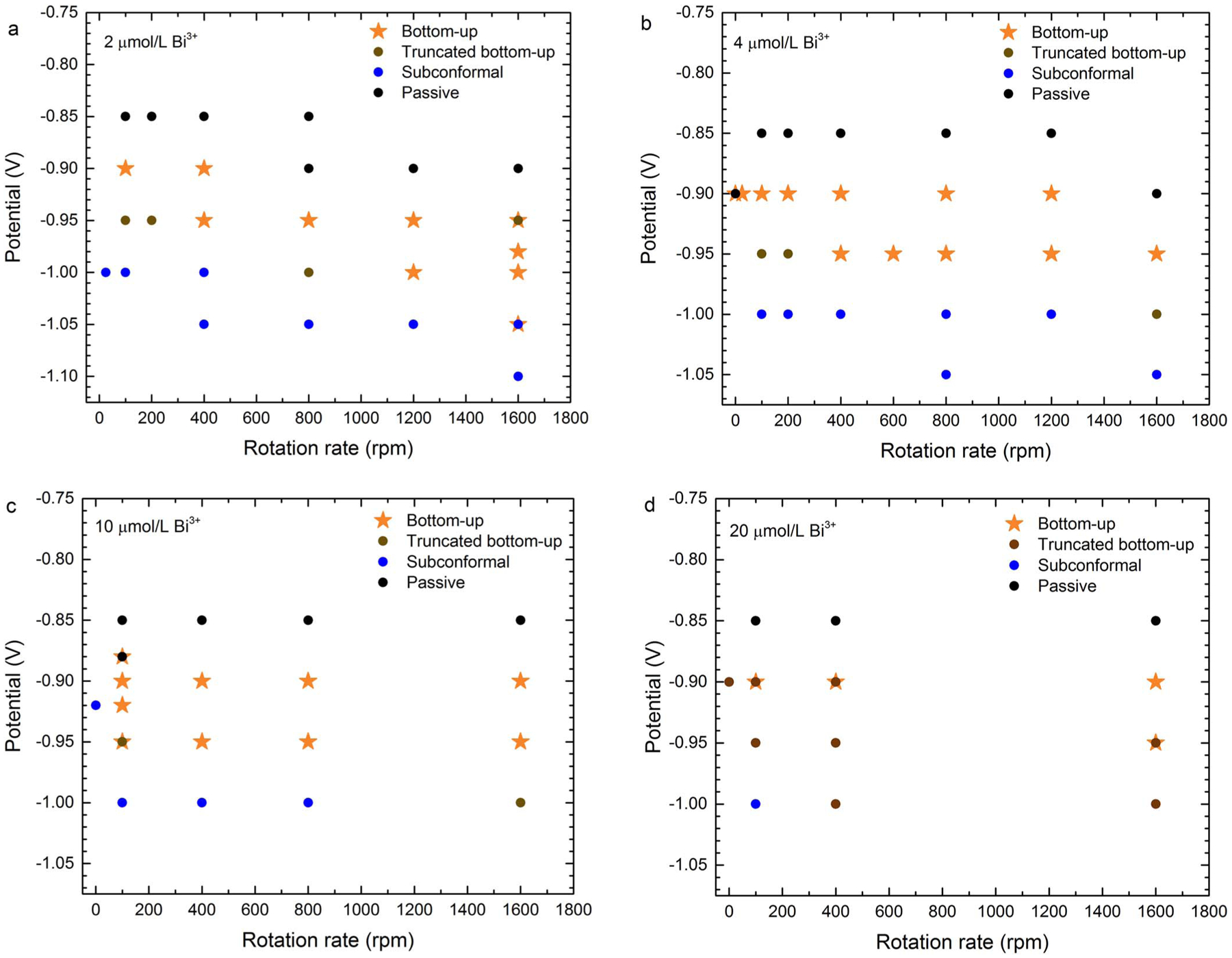
Maps summarizing the results of trench filling experiments over a range of applied potential and substrate rotation rates in electrolyte containing a) 2 μmol/L, b) 4 μmol/L, c) 10 μmol/L and d) 20 μmol/L Bi^3+^.

**Figure 11.**
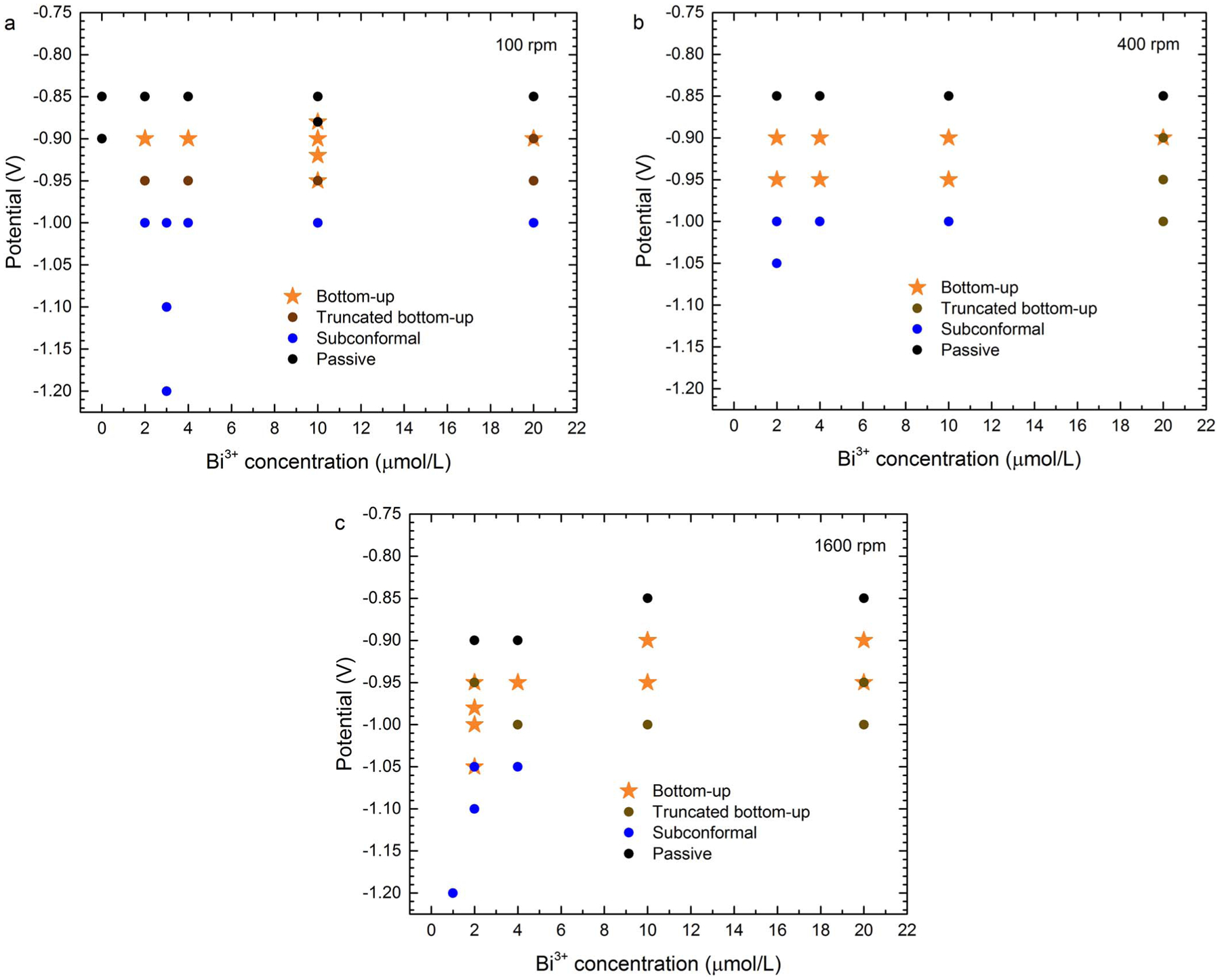
Maps summarizing the results of trench filling experiments over a range of applied potential and Bi^3+^ concentrations at substrate rotation rates of a) 100 rpm, b) 400 rpm and c) 1600 rpm.

The results of partial and full feature filling experiments are summarized as processing parameter maps in Figs. 10 and 11. Feature filling is classified as one of four types: passivated, bottom-up, truncated bottom-up, or subconformal. The first two manifestations are unambiguous. Truncated bottom-up deposition indicates substantial deposition over a portion of the sidewall accompanies enhanced bottom-up deposition of at least 20% of the trench height. This transition zone designation includes two subsets of behavior; one results in void-free, superconformal filling while the other includes minor voids, typically high aspect seams along the center line of the trench. Subconformal filling may exhibit limited bottom-up filling, arbitrarily defined here to be less than 20% of the trench height. Filling for a range of potentials and substrate rotation rates is summarized for different Bi^3+^ concentrations in Fig. 10. Filling over a range of potentials and Bi^3+^ concentrations is summarized for different substrate rotation rates in Fig. 11. As in Fig. 6, fully suppressed surfaces are observed at −0.85 V, and more positive potentials, while subconformal filling is observed at potentials at, or more negative than, −1.00 V. Bottom-up filling is obtained at potentials between these two regimes. The truncated version of bottom-up filling is observed at low rotation rates and potentials between those yielding bottom-up and subconformal deposition (Fig. 10) and is correlated with the loss of suppression on the free surface and sidewalls (Fig. 8).

The truncated bottom-up filling observed for higher Bi^3+^ concentrations at 1600 rpm and an applied potential of −1.00 V (Fig. 11c) is associated with deposition current on the specimen reaching 7 mA, reflecting acceleration enabled by the Bi^3+^ and elevated Au^+^ transport. Based on the 6 Ω impedance measured with the RDE, the actual interface potential is some +40 mV higher due to potential drop across the electrolyte. It is likely that deposition at −1.0 V absent resistive losses would yield subconformal deposition. This would make filling at the higher Bi^3+^ concentrations and rotation rates where truncated bottom-up filling is presently indicated more like that obtained at the lower Bi^3+^ concentrations and rotation rates; i.e., the maps in Figs. 11b and 11c would even more closely resemble that in Fig. 11a.

Before closing this experimental section, it is noted that deposition across approximately 100 μm of the leading edge of each rotating wafer fragment frequently differed from that across the remainder (the “helicopter blade” specimen being 3 mm wide and approximately 8 mm long outside the clamped region). This variation is not detailed further herein. At the pattern length scale, deposition varied for several micrometers from either end of the trench arrays (also not shown), with filling otherwise uniform. Flow direction, which ranged from orthogonal to parallel to the trench arrays across each specimen, yielded no qualitative change of bottom-up filling under planview optical examination (although more subtle variation is possible). The symmetric feature filling evident in the figures was generally observed although for a small number of specimens at the highest rotation rate limited asymmetry across the trench width was observed. In addition, deposition in electrolyte containing 4 μmol/L Bi, based on Bi dissolution charge, subjected to argon sparging prior to deposition and argon injection in the headspace of the cell during deposition exhibited the same behaviors: reduced deposition on the field with increased rotation rate as in Fig. 8 and dependence of filling on applied potential at 400 rpm as shown in Fig. 6.

## Discussion

As shown perhaps most dramatically in Fig. 7, a new process for bottom-up, void-free trench filling with Au has been revealed. That said, much remains to be done to understand the mechanism behind localization of the deposition reaction in feature filling and its unusual dependence on hydrodynamics. Voltammetric studies reveal increased current densities with potential and Bi^3+^ concentration up to 20 μmol/L (Fig. 1). As noted, potential and time dependent accumulation of the dilute Bi^3+^ additive on the deposit surface underlies the increase. Given the dilute nature of the additive its accumulation is initially dependent on the RDE transport conditions. For higher additive concentrations of 10 μmol/L and 20 μmol/L Bi^3+^ the acceleration is such that the metal deposition rate becomes limited by Au(SO_3_)_2_^3−^ transport. Adsorbate deactivation is indicated by the re-assertion of suppression at the most positive potentials in Fig. 5b. A similar process is also present under conditions of active Au deposition as suggested by Fig. 1h but remains unexplored. The incubation period in chronoamperometry (Fig. 3) as well as that which precedes upward filling (Figs. 6 and 7) also remain to be rationalized.

**Figure 12.**
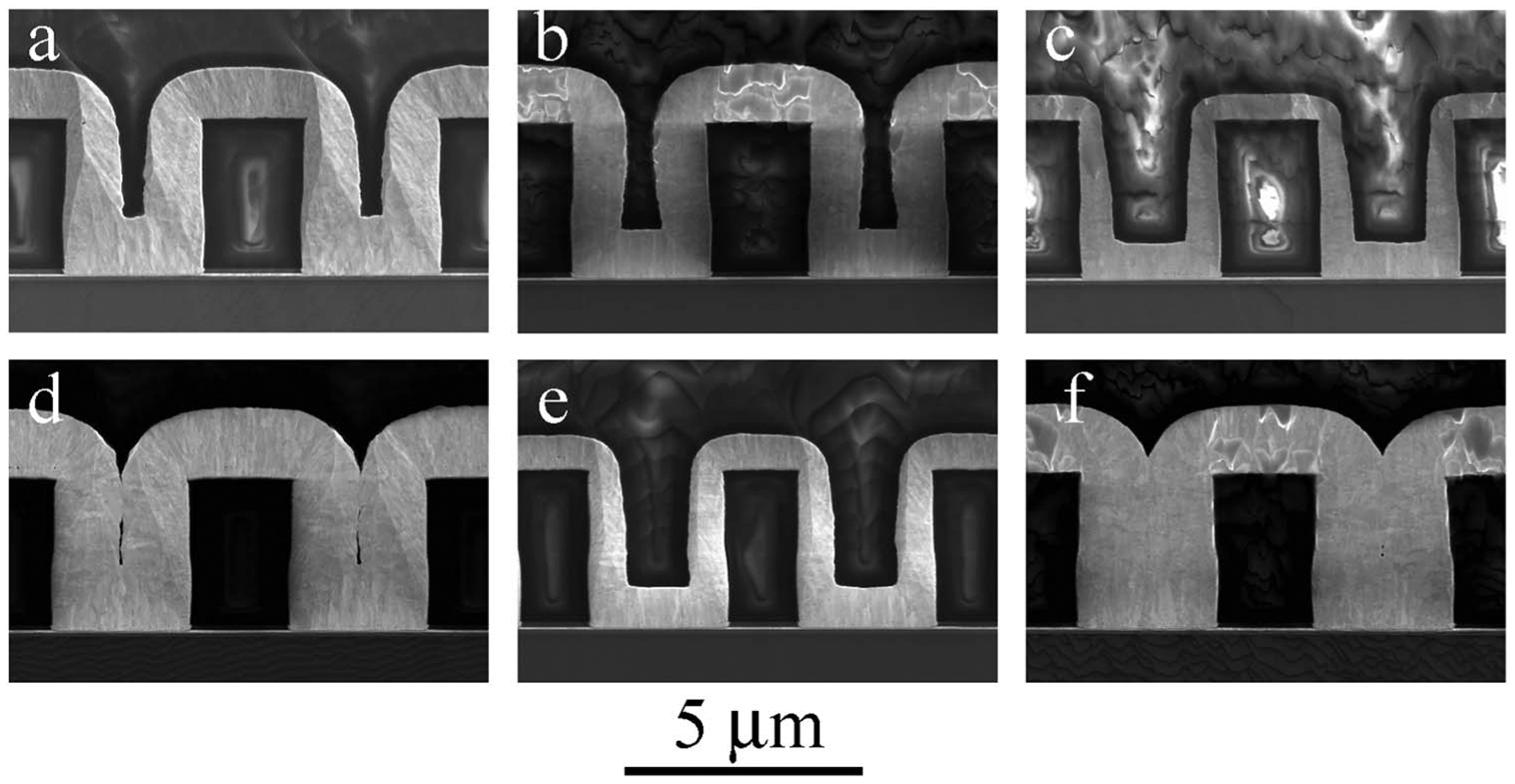
Gold deposition for a) 20 min at −0.95 V in electrolyte containing 2 μmol/L Tl^+^, substrate rotation rate 1600 rpm. b-f) Impact of rotation rate with 6 μmol/L Tl^+^. Rotation rates b) 100 rpm, c-d) 400 rpm and e-f) 1600 rpm. Potentials −0.90 V except for c) at −0.85 V. Deposition times 20 min except for e) at 10 min. Deposition is subconformal at 100 rpm, suggestive of metal ion depletion. It exhibits only a modest enhancement in features filled at higher rotation rates and less negative potential (partly obscured by outward bulging of the nonvertical sidewalls).

Despite the analogous use of an accelerating additive, the filling behavior differs in several key respects from Au,^6–8^ Ag^17–20^ and Cu^2^ superfilling behavior reported in other related and distinct electrolyte systems. These other systems also exhibit an incubation period prior to superconformal feature filling but it is often accompanied by significant conformal growth on all surfaces that underlies modification of local adsorbate coverage through area-change and is central to the CEAC superfill mechanism. Also, accelerated deposition in the previous systems usually initiates at the bottom corners of the feature rather than the entire bottom surface as with Bi (Fig. 7 and Fig. 9). The nontrivial relationship between additive acceleration in electroanalytical measurements and evolution of deposition in patterned features is reinforced by the extremely modest superconformal feature filling obtained when Bi is replaced by Tl, also an accelerator of Au deposition in this potential range,^48^ shown in Fig. 12. Whether due to quantitative differences in additive transport, adsorption, or adsorbate incorporation, or qualitative differences in reaction or surface alloying, the inclusion of accelerators in an electrolyte clearly does not guarantee a particular modality of superconformal feature filling.

The limited irregular deposition evident on the sidewalls for bottom-up growth with Bi, despite the indications of seed layer continuity provided by the results with Tl in Fig. 12, is of concern. Further experiments were undertaken to assess the role, if any, of the sidewall seed layer metallurgy, texture, roughness or possible contamination on the filling evolution with Bi. Several different procedures were employed to build up or clean the Au seed layer prior to feature filling experiments. On some substrates, 90 nm of Au was freshly deposited on the existing seed layer in an electron beam evaporator deposition system (base vacuum *<*2.6 × 10^−5^ Pa, i.e., 2 × 10^−7^ Torr) prior to electrodeposition. This included 30 nm with the Au flux along the substrate normal, to coat both free surface and trench bottom, and 30 nm with the substrate rotated so the flux was 30° between the substrate normal and the sidewall normals, for ≈15 nm on each side-wall. Other substrates were modified using an analogous procedure but first with 20 nm of Ti evaporated along each orientation followed by 40 nm of Au along each orientation. The Ti was used to reactively bury any residual organic material prior to deposition of the fresh vapor deposited Au layer. In yet another variation, the original Au seeded wafer fragments were sputter cleaned using an argon ion beam immediately prior to Au electrodeposition. In all cases localized bottom-up filling was observed after deposition at −0.90 V in 20 μmol/L Bi^3+^ electrolyte for substrate rotation rate of 1600 rpm. The onset of increasing deposition on the free surface and sidewalls at lower substrate rotation rates was also consistent with the results from the original Au seed layer. The thicker Au seed layers did exhibit deposition on the sidewalls at −0.95 V that, while still substantially less than on the bottom, was enhanced relative to that on the as-received Au seed layer; smooth and continuous deposits were evident by 2 min even at 1600 rpm. This yielded truncated bottom-up filling in contrast to the strictly bottom-up filling obtained on the as-received substrates under these conditions. These different growth morphologies under identical deposition conditions are reflected in the summary maps of Figs. 10 and 11. It is clear that the seed layer, which is identical in thickness, orientation and texture on the free surface and trench bottoms, cannot underlie passivation of the free surface obtained at higher rotation rates.

Acceleration of deposition on the free surface at −0.95 V as transport decreases (Fig. 8) is unexpected based on the acceleration induced by the Bi^3+^ in the chronoamperometry at this potential (Fig. 3b) as well as the voltammetry (Fig. 1). If transport is limiting one expects the opposite, deposition enhanced by transport (as in Figs. 12b and 12d with Tl), or, if otherwise, one expects little effect (as in Figs. 12d and 12f with Tl). Interestingly, accelerated deposition on the free surface with decreased transport over the 1600 rpm to 100 rpm interval is consistent with the chronoamperometry at −0.90 V (Fig. 3a) over the same range. Furthermore, the feature filling is not necessarily inconsistent with the voltammetry. Specifically, the 1200 grit paper (≈ 4 μm particle size) yields an RDE with roughness closer to the depth of the trenches than the nanometer scale roughness of the free surface. Enhanced deposition on the RDE might thus reflect accelerated deposition in recesses of the surface roughness analogous to the bottom-up trench filling at less negative potentials and uniformly accelerated deposition as on the patterned features at more negative potentials.

Aspects where deposition suggests the CEAC mechanism include very smooth deposits where the free surface is active (Fig. 6 and Fig. 8), also seen with Tl (Fig. 12), consistent with the CEAC surface stabilization.^23,24^ Some specimens grown under conditions bordering full passivation also exhibited growth limited to the lower corners (not shown). The growth front evolution during bottom-up filling in the sequence captured in Fig. 7 could easily be confused with template or through mask plating despite the fact that both side walls and free surface are well covered with the Au seed-layer. This speaks to the importance of passivation on the sidewalls and free surface and suggests comparison to bottom-up Cu filling of TSV although in that case the mechanistic path is associated with breakdown of the suppressor associated with a co-adsorbed combination of polymeric – halide additive. In the present case the Au deposition itself is intrinsically suppressed analogous to that reported for Au cyanide chemistry. Electroanalytical experiments show that Bi^3+^ addition leads to the lifting of the suppression at potentials at or below −0.95 V in a manner that is consistent with the CEAC mechanism leading to void-free superconformal growth such as that shown in Figs. 6 and 8 (4 μmol/L Bi^3+^, 400 rpm at −0.95 V, same specimen).

The constraint of transport on the metal deposition reaction itself is clear in the electroanalytical measurements. Specifically, the plateau in the voltammetry for 10 μmol/L and 20 μmol/L Bi^3+^ arises from limited Au(SO_3_)_2_^3−^ mass transport: the current density at −0.90 V is at the transport limit for 100 rpm, one-half the transport limit for 400 rpm and one-quarter the transport limit at 1600 rpm (Fig. 1). In contrast, deposition at −0.95 V occurs at nearly three-quarters of the transport limit even at 1600 rpm, and deposition at −1.00 V is at the transport limit for all rotation rates examined. The subconformal sidewall deposits in the truncated bottom-up filling at −0.95 V and the subconformal deposits at −1.00 V in Fig. 6 are consistent with the associated metal ion concentration gradients within the filling features. Localization of deposition to the trenches at −0.90 V and patterning of only a portion of the die surface reduce the average current density and associated gradients outside the trenches on patterned specimens.

Taken together, the results presented here detail a unique process for Au filling of patterned features. The bottom-up filling geometry suggests further exploration of its applicability to the filling of even higher aspect ratio features. Processing parameter maps of the deposition potential, Bi^3+^ concentration and hydrodynamics, in combination with control experiments with the brightening additive Tl^+^, establish the key role of Bi^3+^ in void-free bottom up filling. That said, little is known about the Bi^3+^ precursor or the thermodynamics and kinetics of its reduction. Prior work on both overpotential and underpotential deposition of Bi on Au has largely focused on acid media where Bi^3+^_(aquo)_ species are known. In the present work at pH 9.5 hydrolysis to hydroxide or ox species, and/or even clusters thereof, is expected. In work of McIntyre and Peck^5^ on Au deposition in pH 8 phosphate solution similar transport related anomalies associated with BiO^+^ adsorption, reduction and oxidation on Au were reported in the voltammetric behavior. Further work exploring the surface chemistry, speciation, deposition and dissolution of Bi in the alkaline sulfite media will be necessary to bring insight into the dynamics and mechanism behind the feature filling behavior reported herein.

## Conclusions

Superconformal Au filling of sub-micrometer trenches has been demonstrated using a Bi^3+^ additive in a Na_3_Au(SO_3_)_2+_Na_2_SO_3_ electrolyte. Most importantly, a processing window for exclusive bottom-up feature filling has been identified. More broadly, the exploration of feature filling as a function of Bi^3+^ concentration, potential and hydrodynamics reveals four regimes of feature filling behavior; passivated, bottom-up, truncated bottom-up, and subconformal. Electroanalytical measurements reveal hysteretic voltammetry and rising chronoamperometric transients that, for potentials negative of ≈ −0.93 V, are analogous to those seen for other CEAC superfilling electrolytes. At potentials close to this transition bottom-up filling is observed with a complex dependence on hydrodynamics that calls to question the standard models used to deconvolve mixed control reactions into a simple linear combination of mass transport and interface resistance terms. Further work on the surface and solution chemistry of the Na_3_Au(SO_3_)_2_ + Na_2_SO_3_ and Bi^3+^ additive system is underway.

## Footnotes

1 The use of corporate or product names does not imply NIST endorsement; these details are provided only for accuracy of experimental description.
